# Clinical results after very early, early and late arthroscopic arthrolysis of the knee

**DOI:** 10.1007/s00264-021-05193-0

**Published:** 2021-09-04

**Authors:** Lena Eggeling, Leonard Klepsch, Ralph Akoto, Karl-Heinz Frosch

**Affiliations:** 1 Department of Trauma and Orthopaedic Surgery, Sports Traumatology, BG Hospital Hamburg, Bergedorfer Str. 10, 21033 Hamburg, Germany; 2 Asklepios Clinic St. Georg, Lohmühlenstraße 5, 20099 Hamburg, Germany; 3 Department of Trauma and Orthopaedic Surgery, University Medical Center Hamburg-Eppendorf, Martinistrasse 52, 20246 Hamburg, Germany; 4 University of Witten/Herdecke, Cologne Merheim Medical Center, Cologne, Germany

**Keywords:** Arthroscopic arthrolysis, Conservative therapy, Impaired patient outcome

## Abstract

**Purpose:**

Impaired patient outcome can be directly related to a loss of motion of the knee following surgical procedures. If conservative therapy fails, arthroscopic arthrolysis is an effective procedure to improve range of motion (ROM). The purpose of this study was to evaluate the outcome of patients undergoing very early (< 3 months), early (3 to 6 months), and late (> 6 months) arthroscopic arthrolysis of the knee.

**Methods:**

With a follow-up on average at 35.1 ± 15.2 (mean ± SD, 24 to 87) months, 123 patients with post-operative motion loss (> 10° extension deficit/ < 90° of flexion) were included between 2013 and 2018 in the retrospective study, while eight patients were lost to follow-up. A total of 115 patients were examined with a minimum follow-up of two years. Twenty percent (*n* = 23) of patients of this study population had a post-operative motion loss after distal femoral fracture, 10.4% (*n* = 12) after tibial head fracture, 57.4% (*n* = 66) after anterior/posterior cruciate ligament (ACL/PCL) reconstruction, 8.7% (*n* = 10) after infection of the knee, and 3.4% (*n* = 4) after patella fracture. Thirty-seven patients received very early (< 3 months, mean 1.8 months) arthroscopic arthrolysis, and 37 had early (3 to 6 months, mean 4.3 months) and 41 late (> 6 months, mean 9.8 months) arthroscopic arthrolysis after primary surgery.

**Results:**

The average ROM increased from 73.9° before to 131.4° after arthroscopic arthrolysis (*p* < 0.001). In the group of very early (< 3 months) arthroscopic arthrolysis 76% (n = 28) of the patients had a normal ROM (extension/flexion 0/140°), in the group of early (3–6 months) arthrolysis 68% (n = 25) of the patients and in the group of late arthrolysis 41.5% (n = 17) of the patients showed a normal ROM after surgery (p = 0.005). The total ROM after arthrolysis was also significantly increased in the group of very early and early arthrolysis (136.5° and 135.3° vs. 123.7°, *p* < 0.001). A post-operative flexion deficit occurred significantly less in the group of very early and early arthroscopic arthrolysis compared to the late arthroscopic arthrolysis (3.9° and 4.2° vs. 16.6°, *p* < 0.001). Patients treated with very early (< 3 months) and early (3 to 6 months) showed a significantly increased post-operative Tegner score of 4.8 ± 1 and 4.7 ± 1.1 compared to 3.8 ± 1.1 in the group of late arthroscopic arthrolysis (> 6 months, *p* < 0.001).

**Conclusions:**

An arthroscopic arthrolysis is highly effective and leads to good to excellent mid-term results. An early arthroscopic arthrolysis within 6 months after primary surgery leads to significantly improved ROM and functional scores compared to the late arthrolysis (> 6 months).

## Introduction

As the postoperative loss of motion of the knee, like an extension deficit of more than 5° or a reduced flexion of 110°, is a common complication in various surgical treatments, it may occur in up to 4% after anterior cruciate ligament (ACL) reconstruction [1, 2]. When the ACL reconstruction (ACLR) is combined with an open reconstruction of the medial collateral ligament (MCL), the incidence of post-operative motion loss is even higher with rates up to 13% [1, 2]. Following the surgical treatment of tibial plateau fractures (TPF), the rate of a loss of motion was shown to be 14%, and after revision procedures, it may even raise up to 50% of the cases [1, 3–5].

Impaired patient outcome can be directly related to a loss of motion of the knee following surgical procedures [4]. When conservative therapy fails, there are only a few treatment options like manipulation under anaesthesia (MUA) or arthroscopic arthrolysis in order to improve the range of motion of the knee (ROM) [6–9]. MUA is an option for treating arthrofibrosis in the early post-operative phase within six weeks after prior surgery and can lead to an improved range of motion [10]. The authors recommend establishing adequate patellar mobilization before attempting MUA to prevent damage to the retropatellar surface [8].

While MUA is associated with a few complications like supracondylar femur fractures and an insufficient improvement in ROM, good results are reported following arthroscopic arthrolysis [11–13]. Only a few studies have reported the outcome of patients after arthroscopic arthrolysis, and little is known about the best timing of this procedure [14, 15].

Therefore, the purpose of this study was to evaluate the outcome of patients undergoing very early (< 3 months), early (3 to 6 months), and late (> 6 months) arthroscopic arthrolysis. We hypothesize that an arthroscopic arthrolysis is highly effective and leads to good mid-term results and that very early and early arthroscopic arthrolysis in patients with postoperative motion loss results in a significantly improved ROM and increased functional scores compared to the late arthroscopic arthrolysis.

## Materials and methods

### Patient population

The retrospective cohort study took place at a level one trauma centre from January 2013 to December 2018 and included all patients treated arthrocopically with post-operative loss of motion of the knee.

Inclusion criteria were postoperative loss of motion (> 10° extension deficit/ < 90° of flexion) of the knee when motion failed to improve despite 6 weeks of intense physiotherapy and arthroscopic arthrolysis of post-operative motion loss. Exclusion criteria were active signs of a complex regional pain syndrome (CRPS), previous total knee arthroplasty (TKA), or non-consolidated fractures of the femur or tibia, as well as post-operative malalignment and an isolated cyclops syndrome.

A normal ROM was defined as extension/flexion of 0/140°. Extension deficit or flexion deficit was compared motion loss on the affected side with the normal contralateral leg. With a follow-up on average at 35.1 ± 15.2 (mean ± SD, 24 to 87) months, 123 patients were included in the retrospective study, while eight patients were lost to follow-up. A total of 115 patients were clinically examined with a minimum follow-up of 24 months. Sixty-nine women and 46 men, mean age 36.95 ± 13.4 years (range 18–60), were clinically examined after arthroscopic arthrolysis. Twenty percent (*n* = 23) of patients of this study population had a post-operative motion loss after distal femoral fracture, 10.4% (*n* = 12) after tibial head fracture, 57.4% (*n* = 66) after ACL/posterior cruciate ligament (PCL) reconstruction, 8.7% (*n* = 10) after infection of the knee, and 3.4% (*n* = 4) after patella fracture.

The study design was approved by the local Ethics Committee.

### Surgical technique for arthroscopic arthrolysis

The standard protocol for preoperative physiotherapy was the same for each patient in the individual groups of very early, early, and late arthroscopic arthrolysis. Thus, physiotherapy was carried out three times a week for 30 minutes in each group before arthroscopic arthrolysis.

Patients with post-operative knee stiffness were arthroscopically addressed, and various portals were used according to the pre-operative ROM. To remove scar tissue, a motorized shaver and radiofrequency electrodes were applied. When a loss of flexion was present, patients received an arthroscopic arthrolysis of the anterior compartments and suprapatellar recessus of the knee. Besides the standard arthroscopic portals (anteromedial/anterolateral), also medial and lateral suprapatellar portals as well as posteromedial and posterolateral portals were added as needed.

When an extension deficit was present, posteromedial and posterolateral portals were used to address the scar tissue in the posterior compartments. Even, a dorsal capsulotomy was carried out as needed. A proximalization of the tibial tubercle was performed when the patellar tendon was retracted due to scar tissue, resulting in a patella baja that led to a severe flexion deficit. After surgery, patients were treated with sufficient analgesia and received careful and pain-free physical therapy, also with the use of continuous passive motion (CPM) devices. Partial weight bearing with 10 kg was indicated post-operatively for the time of wound healing, approximately for 14 days with unlimited range of motion. After that, patients were allowed to put more weight on the leg up to full weight bearing. Patients were given oral non-steroidal anti-inflammatory drugs (NSAID) and corticosteroids to reduce the risk of recurrent scar tissue in case they agreed to the treatment.

### Statistical analysis

Thirty-seven patients received very early (< 3 months) arthroscopic arthrolysis, and 37 had early (3 to 6 months) and 41 late (> 6 months) arthroscopic arthrolysis after the primary surgery.

Mean ± standard deviation was used for continuous variables, and the calculation was based on three groups: patients with very early (< 3 months), early (3 to 6 months), and late (> 6 months) arthroscopic arthrolysis. A subgroup analysis was performed to determine correlations between these three groups (Table 1). Mean differences between these three groups were calculated with the unpaired parametric Student’s *t*-test and the Kruskal–Wallis test for non-parametric parameters. Categorical parameters were compared using the chi-square test. In case of small subgroups (*n* < 5), Fisher’s exact test was used for categorical parameters. Statistical analysis was performed using IBM® SPSS® Statistics Version 22. A *p*-value less than 0.05 was considered significant.

**Table 1:** Patient characteristics according to the time of arthroscopic arthrolysis

	**Very early arthroscopic arthrolysis** (< 3 months) (*n* = 37)	**Early arthroscopic arthrolysis** (3–6 months) (*n* = 37)	**Late arthroscopic arthrolysis** (> 6 months) (*n* = 41)	*p*-value
Age (mean ± SD in years)	34.4 ± 13 (18–60)	33.2 ± 14.1 (21–60)	38.2 ± 13.3 (18–60)	0.556
Sex, female (*n*/%)	19/51.4	25/67.6	25/61	0.358
Right knee (*n*/%)	17/45.9	18/48.6	16/39	0.675
BMI (mean ± SD in kg/m^2^)	27.1 ± 3.8 (20–33)	28.2 ± 4.1 (21–32)	26.4 ± 16.4 (19–36)	0.342
Follow-up (months in mean ± SD)	32 ± 6.7 (24–46)	27.7 ± 3.8 (24–37)	36.7 ± 21.3 (24–87)	0.28
Post-operative oral cortisone (*n*/%)	29/78.4	25/67.6	25/61	0.201
Time between primary surgery and arthroscopic arthrolysis (months in mean ± SD)	1.8 ± 0.4 (0–2.8)	4.3 ± 0.8 (3–6)	9.8 ± 50.3 (7–240)	** < 0.001**
Dorsal capsulotomy (*n*/%)	1/2.7	0	4/9.8	0.127
Osteotomy of the tibial tubercle (*n*/%)	2/5.4	0	2/4.9	0.545
Reasons for post-operative motion loss (*n*/%):				
ORIF distal femoral fractures	8/21.6	8/21.6	7/17.1	0.226
ORIF tibial head fractures	2/5.4	3/8.1	7/17.1	
ORIF patella fractures	0	0	4/9.8	
Arthrotomy after knee infection	4/10.8	2/5.4	4/9.8	
Arthroscopic ACL/PCL reconstruction	23/62.2	24/64.9	19/46.3	
*BMI* body mass index, *ORIF* open reduction and internal fixation, *ACL* anterior cruciate ligament, *PCL* posterior cruciate ligament				

## Results

There were no significant differences between the individual groups of very early, early, and late arthroscopic arthrolysis in terms of age, sex, additional procedures like tibial tubercle osteotomy or dorsal capsulotomy, follow-up, and surgical procedures that led to a post-operative motion loss. Patient characteristics are displayed in Table 1. The average ROM increased from 73.9 ± 36.9° pre-operatively to 131.4 ± 14.6° post-operatively (*p* < 0.001). The pre-operative extension deficit significantly decreased from 7.9 ± 10.2 to 0.8 ± 3.3° after arthroscopic arthrolysis (*p* < 0.001). Also, the mean pre-operative flexion deficit significantly decreased from 56.4 ± 37.4 to 8.5 ± 14.3° after arthroscopic arthrolysis (*p* < 0.001). A normal ROM (extension/flexion: 0/140°) of the knee was post-operatively received in 61% (*n* = 70) of the patients. In the group of very early (< 3 months) arthroscopic arthrolysis, 76% (*n* = 28) of the patients had a normal ROM after arthrolysis; in the group of early (3–6 months) arthrolysis, 68% (*n* = 25) of the patients did not show an extension or flexion deficit, and in the group of late arthrolysis, 41.5% (*n* = 17) of the patients showed a normal ROM after surgery. This difference was statistically significant (*p* = 0.005).

**Fig. 1:**
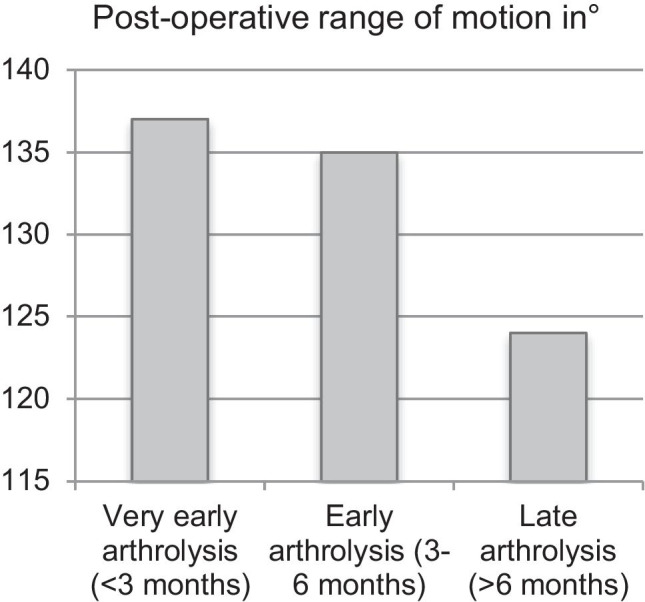
The post-operative range of motion of the knee in degree in regards to the very early, early, and late arthroscopic arthrolysis

**Fig. 2:**
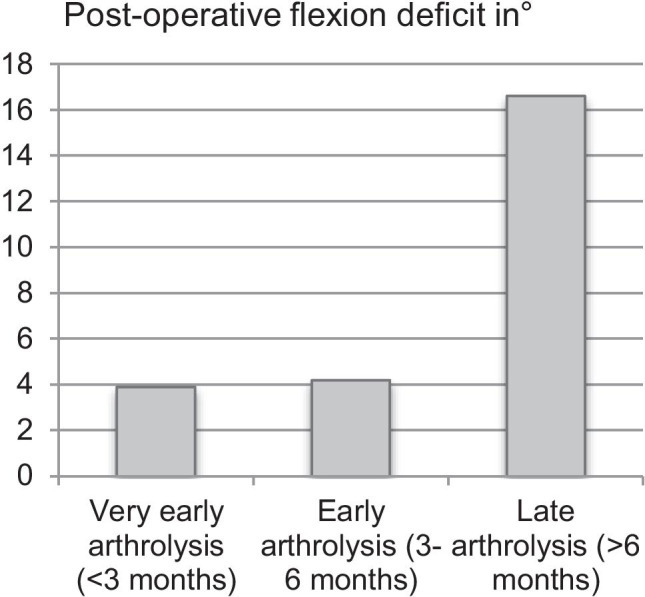
The postoperative flexion of the knee in degree deficit according to the very early, early, and late arthroscopic arthrolysis

The total ROM after arthrolysis was significantly increased in the group of very early and early arthrolysis (136.5° and 135.3° vs. 123.7°, *p* < 0.001, Fig. 1). In the subgroup analysis, it was also shown that a post-operative flexion deficit occurred significantly less in the group of very early and early arthroscopic arthrolysis compared to the late arthroscopic arthrolysis (3.9° and 4.2° vs. 16.6°, *p* < 0.001, Fig. 2). The post-operative extension deficit was significantly reduced in the group of very early arthroscopic arthrolysis (*p* = 0.046), while in the pre-operative assessment, the group of very early arthrolysis showed a significantly increased extension deficit (11.6° vs. 6.5° and 5.9°m *p* = 0.009). The mean Tegner score significantly increased in all patients after arthrolysis compared to pre-operatively (3.2 ± 1.1 vs. 4.4 ± 1.1, *p* < 0.001). Patients treated with very early (< 3 months) and early (3 to 6 months) arthrolysis showed significantly increased post-operative functional scores; thus, the Tegner score was 4.8 ± 1 and 4.7 ± 1.1 in the very early and early arthrolysis compared to 3.8 ± 1.1 in the group of late arthroscopic arthrolysis (> 6 months, *p* < 0.001). The evaluation of the pre- and post-operative parameters is shown in Table 2. There were no post-operative complications within the time of follow-up.

**Table 2:** Evaluation of the pre- and postoperative parameters of the knee compared to the time of arthroscopic arthrolysis

	**Very early arthroscopic arthrolysis** (< 3 months) (*n* = 37)	**Early arthroscopic arthrolysis** (3–6 months) (*n* = 37)	**Late arthroscopic arthrolysis** (> 6 months) (*n* = 41)	*p*-value
Pre-operative extension deficit (° in mean ± SD)	11.6 ± 11 (0–35)	6.5 ± 11.1 (0–40)	5.9 ± 7.7 (0–30)	**0.009**
Post-operative extension deficit (° in mean ± SD)	0 (0)	0.9 ± 5.5 (0–20)	0.5 ± 1.7 (0–5)	**0.046**
Pre-operative flexion deficit (° in mean ± SD)	63.2 ± 35 (10–120)	48.4 ± 36.2 (10–110)	57.8 ± 58.1 (10–110)	0.208
Post-operative flexion deficit (° in mean ± SD)	3.9 ± 6.6 (0–20)	4.2 ± 6.8 (0–20)	16.6 ± 20 (0–80)	** < 0.001**
Pre-operative range of motion (° in mean ± SD)	64.4 ± 35.5 (20–130)	85 ± 34.1 (30–130)	72 ± 39.5 (30–135)	0.163
Post-operative range of motion (° in mean ± SD)	136.5 ± 6.7 (120–140)	135.3 ± 8.2 (120–140)	123.7 ± 20.3 (60–140)	** < 0.001**
Normal range of motion (Ex/Flex 0/140°, *n*/%)	28/75.7	25/67.6	17/41.5	**0.005**
Pre-operative Tegner (points in mean ± SD)	3 ± 1.2 (1–5)	3.5 ± 1 (2–5)	3.1 ± 1.2 (1–5)	0.263
Post-operative Tegner (points in mean ± SD)	4.8 ± 1 (3–7)	4.7 ± 1.1 (3–7)	3.8 ± 1.1 (1–6)	** < 0.001**

*Ex* extension, *Flex* flexion

## Discussion

The main findings of this study are that an arthroscopic arthrolysis is highly effective and leads to good to excellent mid-term results with a mean increase of the ROM of 58°. Further, an early arthroscopic arthrolysis (< 6 months) leads more often to a normal post-operative ROM, an increased total ROM, and a lower flexion deficit after primary post-operative motion loss of the knee compared to the late arthroscopic arthrolysis (> 6 months).

It is commonly known that persistent post-operative knee stiffness results in impaired patient-related outcome [4, 16, 17]. Previous studies demonstrated that impaired post-operative functional scores correlated with a post-operative extension and flexion deficit in primary and revision osteosynthesis of tibial head fractures [3, 5]. Also, Mayr et al. showed that patients with post-operative motion loss suffered from long-term complications: thus, nearly 80% of their patients with arthrofibrosis showed signs of osteoarthritis in the femoro-tibial joint five years after arthroscopic arthrolysis [14]. In most of the cases, signs of osteoarthritis were observed when a persisting flexion deficit occurred. An arthroscopic dorsal capsulotomy was not performed in the aforementioned study, and similar results in post-operative ROM could not be achieved compared to our study. This might be due to the delayed timing of the arthrolysis after primary surgery. Nonetheless, it can be stated that patients in these studies with a motion loss of the knee had an improved patient-related outcome after arthroscopic arthrolysis when the ROM was increased. In this study the Tegner score significantly raised from 3.2 before arthrolysis to 4.4 after arthrolysis, while the ROM improved from 73.9° pre-operatively to 131.4° post-operatively. It can be stated that an improved ROM of the knee correlates with an improved patient-related outcome [18].

In general in post-operative motion loss of the knee, especially in post-TKA arthrofibrosis, the MUA is recommended within two to three months after prior surgery [10, 19–21]. If the range of motion has not been improved, arthroscopic arthrolysis is recommended two to three months after prior surgery. In our patient population, there were a lot of patients with post-operative motion loss after ligament reconstruction. In particular after arthroscopic ligament surgery, there might be also further mechanical reasons to show a post-operative motion loss like cyclops syndrome or cartilage fragments. This is the reason why we indicated arthroscopic arthrolysis at an earlier stage when conservative treatment failed to improve the range of motion of the knee.

Arthroscopic arthrolysis is a valid option to treat post-operative motion loss of the knee when conservative therapy fails, even in the early post-operative phase [13–15, 22]. In this study, there were no patients with a prior TKA, and all of the patients had an arthroscopic arthrolysis after failed conservative treatment to improve the range of motion of the knee. LaPrade et al. showed that arthroscopic arthrolysis improved the overall knee motion from an average ROM of 102 to 129° and that the arthroscopic posteromedial capsular release is effective in addressing symptomatic knee extension deficits [23].

Nevertheless, arthroscopic arthrolysis can also be beneficial for patients suffering from motion loss following total knee replacement (TKA) [24]. Hegazy and Elsoufy showed that the average Knee Society Score improved from 68 points preoperatively to 86 and the average pain scores improved from 30 points pre-operatively to 41 at the time of final follow-up in eight patients following arthroscopic arthrolysis after TKA [24].

Our results are in line with the aforementioned studies, as patients with post-operative motion loss of the knee benefit from arthroscopic arthrolysis as the motion range of the knee and functional scores significantly improve after arthrolysis. An extension and flexion deficit can be effectively treated with an arthroscopic arthrolysis. However, this is the first study to show that the timing of the arthrolysis plays a significant role in the treatment of post-operative motion loss of the knee. Little is known about the optimal timing of the arthroscopic arthrolysis after primary surgery. The authors have suggested performing an arthroscopic arthrolysis within one year after primary surgery [14]. A previous study showed the results of a surgical arthroscopic lysis of knee adhesions at a mean time of 244 days/8 months between osteosynthesis and arthrolysis [25]. The pre-operative ROM significantly increased from 73 to 104° at a mean follow-up time of 135 days. The inferior results of the achieved post-operative ROM compared to this study could be associated with the delayed timing of the arthrolysis. This presenting study could point out that there is a correlation between the post-operative ROM and functional scores and the timing of the arthrolysis. Thus, patients with very early (< 3 months) and early (3 to 6 months) arthroscopic arthrolysis showed significant increased ROM of the knee and functional scores compared to the late (> 6 months) arthrolysis. The post-operative ROM of patients with very early and early arthrolysis (< 6 months) was 136.5° and 135.3° compared to the late arthrolysis of 123.7°. Also, the post-operative flexion deficit was significantly reduced in the group of the very early and early arthroscopic arthrolysis (3.9° and 4.2° vs. 16.6°), while the difference in the post-operative extension deficit between the groups was clinically not relevant. Further, a normal ROM in the group of very early and early arthrolysis was received in 76% and 68% of the patients compared to 41.5% of patients after late arthroscopic arthrolysis. Also, the post-operative Tegner score was significantly increased in the group of patients with very early and early arthrolysis (4.8 and 4.7 points) compared to the late arthrolysis (3.8 points). While there are only small differences in the group of very early and early arthrolysis, we would recommend performing an arthroscopic arthrolysis within six months after primary surgery. To the best of our knowledge, there is no previous study that analyzed the correlation between the timing of arthroscopic arthrolysis and the post-operative outcome of the patients. Our results indicate that an early arthroscopic arthrolysis within six months after primary surgery leads to significantly improved ROM and functional scores compared to the late arthrolysis. Patients benefit from an early arthroscopic arthrolysis less than six months.

Complications after arthroscopic arthrolysis are rare. A systematic review of arthroscopies in symptomatic patients after TKA revealed a complication rate of only 0.5%, even though most of the studies reported no complications after arthroscopic procedures [26]. Although in this study there were also no complications related to arthroscopic arthrolysis at the time of follow-up, this procedure is not risk-free. Infection, remaining or recurrent flexion or extension deficit, damage to cartilage, meniscus, and vascular/nerve bundle are potential risks after arthroscopic arthrolysis [27, 28].

There are a few limitations in this study. As the follow-up period was at mean 35 months, post-operative long-term complications like osteoarthritis could not be observed. Also, the study population consists of a very inhomogeneous group of patients, as different pre-operative diagnoses were present and the possible impact on the results might be underestimated. Another limitation is that in the group of “late arthrolysis,” the range of times between surgery and arthroscopic arthrolysis is vast (7–240 months). Further limitations of this study are the retrospective study design, the relatively small sample size, and the lack of randomization or mixed pair analysis.

Also, this study did not include microbiological or histological results; thus, it is unclear whether a possible bias regarding post-operative infection of the knee altered the results.

## Conclusions

An arthroscopic arthrolysis is highly effective and leads to good to excellent mid-term results. Also, this is the first study to show that an early arthroscopic arthrolysis within six months after primary surgery leads to significantly improved ROM and functional scores compared to late arthroscopic arthrolysis (> 6 months).

## Data Availability

Our manuscript has associated data in a data repository.
